# Effects of Isotemporal Substitution Analysis of Sedentary Behavior, Sleep, and Physical Activity on Physical Performance in Older Adults

**DOI:** 10.1007/s10935-026-00904-y

**Published:** 2026-03-04

**Authors:** Matheus Marques e Marques, Antônio Ribeiro Neto, Leandro Alonso do Espírito Santo, Natália Lujan Ferraz, Ricardo Ansaloni Oliveira, Joilson Meneguci, Jeffer Eidi Sasaki, Jair Sindra Virtuoso Júnior

**Affiliations:** 1 Federal University of Triângulo Mineiro (UFTM), Uberaba, MG Brazil; 2 Department of Sport Sciences, Federal University of Triângulo Mineiro (UFTM), Uberaba, MG Brazil

**Keywords:** Aging, Functional performance, Physical activity, Sedentary time, Sleep

## Abstract

Analyze the effects of substituting time spent in sleep, sedentary behavior, and moderate-to-vigorous physical activity on the physical performance of older adults. The sample consisted of 457 participants from the Alcobaça Elderly Health Longitudinal Study. Physical performance was measured using the Short Physical Performance Battery, while physical activity and sedentary behavior were assessed with the International Physical Activity Questionnaire, and sleep with the Pittsburgh Sleep Quality Index. For statistical analysis, the isotemporal substitution approach was applied to examine the hypothetical effects of reallocating time between sleep, sedentary behavior, and moderate-to-vigorous physical activity on physical performance. The results showed that substituting small periods of sleep or sedentary behavior with moderate-to-vigorous physical activity was associated with a significant reduction in the risk of poor physical performance. The reallocation of just 5 min/day already showed a protective effect (a reduction of 13% to 14%), with an increasing impact proportional to the amount of time replaced, reaching an 82% reduction for 60 min/day. Model-based analyses suggest that replacing short periods of sedentary behavior or sleep with moderate-to-vigorous physical activity is associated with a lower risk of poor physical performance in older adults.

## Introduction

The population has been aging substantially, and it is expected that by 2050, 22% of the world’s inhabitants will be older adults (Kanasi et al., 2016). In Brazil, projections indicate that by 2060, about one-quarter of the population (25.5%) will be elderly (Instituo Brasileiro de Geografia e Estatística., 2024). Along with aging, there is a yearly decline of 1% to 3% in different systems, particularly from the fifth decade of life onward (He et al., 2018), associated with reductions in physical performance in terms of muscle strength, balance, and gait (Cooper et al., 2011).

Physical performance is defined as the ability to integrate physiological mechanisms into coordinated movements in order to perform a physical function, that is, the ability to carry out tasks such as sitting down and standing up from a chair (Cress et al., 1996; Freiberger et al., 2012), for example. The assessment of physical performance can help identify potential risks or early stages of functional decline (Fátima Ribeiro Silva et al., 2021).

Regular practice of moderate-to-vigorous physical activity (MVPA) has been shown to be a protective factor against poor physical performance in older adults (Rogers et al., 2014, 2017). On the other hand, even physically active individuals who remain in prolonged sedentary behavior (SB)—defined as activities performed in a sitting/reclining posture with energy expenditure below resting values of ≤ 1.5 METs (Tremblay et al., 2017), —are still subject to adverse health effects (Katzmarzyk et al., 2019; Pinto et al., 2023; Song et al., 2015).

The prevalence of physical inactivity among older adults ranges from 18.5%, 33%, and 50.4%, according to different studies (Muhl et al., 2020; Peixoto et al., 2018; Soares et al., 2024). Moreover, about 60% report sitting for more than 4 h daily and 67% for more than 8.5 h during waking hours (Harvey et al., 2013). Understanding the relationship between physical activity (PA), SB, and physical performance is crucial, since low levels of performance are associated with greater disability and mortality in older adults (Pavasini et al., 2016).

Sleep can also negatively influence muscle strength and contribute to the development of different adverse health conditions (Bohannon, 2019), for example. Older adults may present reduced handgrip strength when associated with poorer sleep quality (Li et al., 2021) and worse overall physical performance (Denison et al., 2021).

Few studies have demonstrated the relationship between reallocations of different behaviors and their effects on physical performance; furthermore, none have taken sleep time into account. Therefore, the aim of this study was to examine the hypothetical effects of reallocating sleep and SB time to MVPA on the physical performance of older adults.

## Methods

### Study Design

This study is part of the Alcobaça Elderly Health Longitudinal Study (ELSIA), characterized as an observational and cross-sectional study conducted with the aim of understanding the living conditions of older adults residing in the municipality of Alcobaça, located in the southern region of Bahia, Brazil. Alcobaça is a small coastal municipality with an estimated population of approximately 20,000 inhabitants, marked by socioeconomic diversity and with a significant proportion of older adults, making it a relevant setting for research on aging and health.

## Participants

The initial population consisted of 743 older adults registered in the Family Health Strategy. Of these, 158 were not located, 54 refused participations, 58 were excluded, and 16 did not complete the battery of physical tests, resulting in a final sample of 457 individuals.

Exclusion criteria included: a score < 12 on the Mini-Mental State Examination adapted for Brazil (Almeida, 1998; Brucki et al., 2003), significant loss of visual or hearing acuity, use of a wheelchair, severe sequelae of stroke, or terminal stage of disease.

Data collection was conducted in two stages: (1) an individual interview using a multidimensional questionnaire containing sociodemographic, behavioral, and health information; and (2) the administration of physical tests to assess performance and measure levels of PA and SB.

## Sociodemographic Variables

Sociodemographic variables and health indicators were obtained through a semi-structured questionnaire and included the following items: sex (male or female), age group (60–69 years, 70–79 years, or 80 years and older), marital status (living with a partner or not), education level (illiterate, 1–4 years of schooling, or more than 5 years of schooling), household arrangement (living alone, with a spouse or other, or in an extended family), depressive symptoms (absent or present), and polypharmacy (taking no medications, 1–4 medications, or more than 5 medications).

## PA and SB

To assess PA levels, the long version of the International Physical Activity Questionnaire (IPAQ) was used, validated for Brazilian older adults (Benedetti et al., 2004, 2007). The instrument considers moderate- and vigorous-intensity activities performed in bouts of at least 10 consecutive minutes across the domains of work, leisure, transportation, and household activities. Weekly PA time was calculated by summing up the minutes spent in moderate activity and twice the minutes spent in vigorous activity. Based on this, participants were classified as insufficiently active (< 150 min/week of MVPA) or sufficiently active (≥ 150 min/week of MVPA).

SB was estimated as daily time spent sitting during activities such as work, transportation, reading, and resting, considering both weekdays and weekends. The weighted average was calculated using the formula: [(sitting time on weekdays × 5) + (sitting time on weekends × 2)] ÷ 7. The 75th percentile of the sample (≥ 540 min/day) was adopted as the cutoff point to classify excessive SB (Santos et al., 2017). Older adults with higher levels of SB (≥ 75th percentile) are more likely to present depressive symptoms when compared to those who meet PA recommendations (Santos et al., 2017).

## Physical Performance

Physical performance was assessed using the Brazilian version of the Short Physical Performance Battery (Guralnik et al., 1994). This battery is composed of a balance test, a gait speed test, and a chair stand test. Each test is scored from 0 to 4 points, and the final score is obtained by summing the scores of the three tests, resulting in a total score ranging from 0 to 12 points. Participants scoring 0–6 points were classified as having poor physical performance, while those scoring 7–12 points were classified as having good physical performance (Guralnik et al., 1994).

### Sleep Assessment

To assess sleep quality, the Brazilian version of the Pittsburgh Sleep Quality Index (Bertolazi et al., 2011) was used. After completing all questions, the last item of the questionnaire was used to obtain the time spent sleeping by the older adult. The question was asked as follows: “During the past month, how many hours of sleep did you get per night?” For data analysis, the answer was converted into minutes to allow for time reallocation in the analysis.

### Data Analysis

The database was created using Epidata 3.1b with double data entry, and analyses were performed using SPSS 25.0. Descriptive statistics were used to characterize the sample with absolute and relative frequencies, means, and measures of dispersion.

To evaluate the effects of reallocating time between sleep, SB, and MVPA on physical performance, the isotemporal substitution approach was used (Mekary & Ding, 2019). In this method, activity times are divided into time units (5 to 60 min) and summed to create a “total behavior” variable, which represents the finite amount of time available in a day.

All activity components are entered simultaneously into the regression model together with the total behavior variable. Hypothetical substitution is operationalized by intentionally removing one activity from the model while keeping the total behavior constant. Under this specification, the regression coefficients of the remaining activities can be interpreted as the estimated effects of reallocating time from the excluded activity to the included activity, minute by minute, while holding the other behaviors and covariates constant.

Analyses were conducted using Poisson regression with robust variance, expressed as prevalence ratios (PR) and 95% confidence intervals (CI). All models were adjusted for age group, polypharmacy, hospitalization, depressive symptoms, and marital status.

## Results

The mean age of the 457 participants was 70.14 ± 8.21 years, with 37.6% male, 46.8% married, 35.8% functionally illiterate, 83.6% reporting no hospitalizations, 88.6% without depressive symptoms, 55.6% living in multigenerational households, and 61.5% taking 1–4 different medications (Table 1). The characterization of sociodemographic variables and health indicators according to physical performance is presented in Table 1.

**Table 1:** Sociodemographic characteristics and health indicators according to physical performance

Variables	Total	Good Performance	Poor Performance	*P* (X²)
Sex				0.306
Male	172 (37.6%)	153 (38.55%)	19 (31.7%)	
Female	285 (62.4%)	244 (61.5%)	41 (68.35)	
Age				0.001*
60–69 years	254 (55.6%)	243 (95.75%)	11 (4.3%)	
70–79 years	135 (29.5%)	117 (86.7%)	18 (13.3%)	
≥ 80 years	68 (14.39%)	37 (54.4%)	31 (45.65)	
Marital Status				0.001*
Single/Separated/Divorced	121 (26.5%)	108 (89.35)	13 (10.7%)	
Married	214 (45.85)	196 (91.6%)	18 (8.4%)	
Widowed	122 (26.7%)	93 (76.2%)	29 (23.8%)	
Education				0.204
Illiterate	147 (32.3%)	124 (84.4%)	23 (15.6%)	
1–4 years	163 (35.8%)	140 (85.9%)	23 (14.1%)	
≥ 5 years	145 (31.9%)	132 (91%)	13 (9%)	
Hospitalization				0.002*
Yes	75 (16.4%)	57 (76%)	18 (24%)	
No	382 (83.65)	340 (89%)	42 (11%)	
Depressive Symptoms				0.024*
Absent	405 (88.6%)	357 (88.1%)	48 (11.9%)	
Present	52 (11.4%)	40 (76.95)	12 (23.1%)	
Household Arrangement				0.587
Living Alone	74 (16.2%)	66 (89.2%)	8 (10.8%)	
With Spouse/Others	129 (28.22%)	114 (88.4%)	15 (11.6%)	
With Children/Grandchildren	254 (55.6%)	217 (85.45)	37 (14.6%)	
Medication Use				0.018*
None	98 (21.4%)	92 (92.9%)	6 (6.1%)	
1–4 medications	281 (61.5%)	243 (86.5%)	38 (13.5%)	
≥ 5 medications	78 (17.1%)	62 (79.5%)	16 (20.55)	

*X²: chi-square

In the isotemporal substitution models (Table 2), reallocating time from sleep or SB to MVPA was significantly associated with better physical performance. The larger the amount of time reallocated, the stronger the protective effect observed. Specifically, replacing 5 min/day of SB with MVPA was associated with a 13% lower prevalence of poor physical performance (PR = 0.87), increasing to a 82% lower prevalence when the substitution involved 60 min/day (PR = 0.18).

**Table 2:** Isotemporal substitution models of the association between reallocating time in sleep, SB, and MVPA with physical performance

Substitution Model	Sleep PR (95% CI)	SB PR (95% CI)	MVPA PR (95% CI)
5 min			
Sleep Substitution	-	1.00 (0.99–1.01)	0.87 (0.76–0.98)*
SB Substitution	0.99 (0.98–1.00.98.00)	-	0.86 (0.76–0.98)*
10 min			
Sleep Substitution	-	1.00 (0.99–1.03)	0.75 (0.59–0.97)*
SB Substitution	0.99 (0.97–1.01)	-	0.75 (0.58–0.97)*
15 min			
Sleep Substitution	-	1.00 (0.98–1.03)	0.66 (0.45–0.95)*’
SB Substitution	0.99 (0.97–1.01)	-	0.65 (0.44–0.95)*
20 min			
Sleep Substitution	-	1.01 (0.96–1.06)	0.57 (0.35–0.94)*
SB Substitution	0.98 (0.94–1.03)	-	0.56 (0.34–0.94)*
25 min			
Sleep Substitution	-	1.01 (0.96–1.07)	0.50 (0.26–0.93)*
SB Substitution	0.98 (0.92–1.04)	-	0.49 (0.26–0.92)*
30 min			
Sleep Substitution	-	1.02 (0.95–1.09)	0.43 (0.20–0.91)*
SB Substitution	0.97 (0.91–1.04)	-	0.42 (0.19–0.91)*
35 min			
Sleep Substitution	-	1.02 (0.94–1.10)	0.38 (0.16–0.90)*
SB Substitution	0.97 (0.90–1.05)	-	0.37 (0.15–0.90)*
40 min			
Sleep Substitution	-	1.02 (0.93–1.12)	0.33 (0.12–0.89)*
SB Substitution	0.97 (0.88–1.06)	-	0.37 (0.11–0.88)*
45 min			
Sleep Substitution	-	1.03 (0.93–1.14)	0.28 (0.09–0.88)*
SB Substitution	0.96 (0.87–1.07)	-	0.27 (0.08–0.87)*
50 min			
Sleep Substitution	-	1.03 (0.92–1.15)	0.25 (0.07–0.86)*
SB Substitution	0.96 (0.86–1.08)	-	0.24 (0.06–0.86)*
55 min			
Sleep Substitution	-	1.03 (0.91–1.17)	0.21 (0.05–0.85)*
SB Substitution	0.96 (0.85–1.09)	-	0.20 (0.05–0.85)*
60 min			
Sleep Substitution	-	1.04 (0.91–1.19)	0.19 (0.04–0.84)*
SB Substitution	0.95 (0.83–1.09)	-	0.18 (0.04–0.83)*

*CI* Confidence Interval, *PR* Prevalence Ratio, *SB* Sedentary Behavior, *MVPA* Moderate-to-Vigorous Physical Activity. PR was adjusted for age group, hospitalization, polypharmacy, depressive symptoms, and marital status

## Discussion

The aim of the present study was to understand how reallocating time from SB and sleep to MVPA may influence physical performance. The results showed that the greater the substitution of SB and sleep (5 to 60 min) with MVPA, the stronger the protective effect observed (13% to 82%) against poor physical performance in older adults, in a directly proportional manner to the amount of time reallocated.

Regarding demographic characteristics, poorer physical performance was more frequently observed among older participants, those aged 80 years and over, widowed individuals, participants with a history of hospitalization, depressive symptoms, and higher medication use. These findings are consistent with previous evidence showing that advanced age, multimorbidity, depressive symptoms, and polypharmacy are important determinants of functional decline in older adults. Such characteristics may increase vulnerability to reduced mobility, balance impairments, and lower muscle strength, thereby influencing physical performance outcomes (Cooper et al., 2011a; Nicholson et al., 2024; Pavasini et al., 2016). Therefore, demographic and health-related factors should be considered when interpreting the associations between time reallocations and physical performance, as they may modulate the magnitude of these relationships.

Low physical performance is an important risk factor for falls among community-dwelling older adults (Welch et al., 2021). Declines in physical performance begin gradually, mainly due to the accumulation of comorbidities, highlighting the importance of increasing the time spent both in light-intensity activities and in MVPA, to mitigate these losses (Bann et al., 2015; Ma et al., 2021).

The findings of this study demonstrate that reallocating just 5 min of sleep or SB to 5 min of MVPA already increases the protective factor against poor performance by 13% and 14%, respectively. PA plays a crucial role in minimizing both the physiological changes associated with aging and the comorbidities that may be linked to SB (Seals et al., 2016).

Reducing SB in the daily activities of older adults is one of the main strategies to preserve muscle strength and physical function (Lai et al., 2023). Potential favorable effects have been identified even with the substitution of only 10 min/day of SB with MVPA (Yasunaga et al., 2017). These findings indicate that small changes in the daily routine of older adults may lead to significant gains in physical performance.

Sleep, muscle strength (Denison et al., 2021; Pana et al., 2021), and physical performance (Chien & Chen, 2015; Denison et al., 2021) may interact and influence one another throughout the aging process. The present study showed that the greater the substitution of sleep time with MVPA, the stronger the protective effect observed. However, this reallocation should be carefully considered, since recommendations for older adults suggest 7 to 8 h of sleep per day (Chaput et al., 2020), and any reduction may contribute to the development of adverse health conditions and poorer physical performance (Denison et al., 2021). In this context, reallocating time from SB to MVPA is advisable, given that older adults frequently experience sleep disturbances (Neikrug & Ancoli-Israel, 2010).

Recent evidence indicates that the association between sleep duration and frailty in older adults follows a non-linear pattern, in which both short and long sleep durations are related to a higher risk of functional decline (Liu et al., 2025; Qian et al., 2025). In contrast, analyses based on isotemporal substitution models show that reallocating time from SB to PA, particularly of moderate intensity, is consistently associated with lower levels of frailty, better physical performance, and a reduced risk of functional disability (Martins et al., 2023; Schmidt et al., 2025). In this context, time-reallocation strategies should prioritize reducing SB in favor of PA, with substitution of sleep time being recommended only in cases of excessive sleep duration beyond general guidelines.

The isotemporal substitution model represents a realistic approach and is considered a gold-standard mathematical method for reallocating time from one activity to another. Nevertheless, it cannot replace experimental evidence (Mekary & Ding, 2019; Sánchez-Sánchez et al., 2019). Furthermore, the present study has an observational cross-sectional design, which may lead to issues related to reverse causality, thereby limiting the ability to establish causal relationships between the variables.

Additionally, the use of self-reported measures of PA and SB (IPAQ) and sleep (PSQI) represents a recognized methodological limitation, as these instruments are subject to recall and reporting bias, which may result in overestimation of PA, underestimation of SB, and imprecision in sleep assessment. However, it should be noted that the IPAQ and the PSQI are widely used and validated instruments in population-based studies involving adults and older adults, which supports comparability with previous investigations. Moreover, because such biases tend to be systematic, they are more likely to affect absolute estimates of reported time than the relative associations between behaviors and physical performance, which are the primary focus of the present study. Thus, although this limitation should be considered when interpreting the findings, it is believed that it does not compromise the direction or consistency of the observed associations.

## Conclusion

The model-based results suggest that the substitution of short periods of sleep or SB with MVPA may be associated with a lower risk of poor physical performance in older adults. These findings indicate the potential relevance of reducing SB and encouraging regular PA as a simple strategy to support healthier aging.

These findings suggest that replacing short periods of SB with MVPA, even in small daily amounts, represents a simple, feasible, and effective strategy to preserve physical performance in older adults, with potential applicability in clinical recommendations and health promotion initiatives.
